# A Bone Morphogenetic Protein (BMP)-derived Peptide Based on the Type I Receptor-binding Site Modifies Cell-type Dependent BMP Signalling

**DOI:** 10.1038/s41598-019-49758-x

**Published:** 2019-09-17

**Authors:** Zhen Tong, Jingxu Guo, Robert C. Glen, Nicholas W. Morrell, Wei Li

**Affiliations:** 1 The Department of Medicine, University of Cambridge School of Clinical Medicine, Cambridge, CB2 0QQ United Kingdom; 2 Centre for Molecular Informatics, Department of Chemistry, University of Cambridge, Cambridge, CB2 1EW United Kingdom; 3 Department of Metabolism, Digestion and Reproduction, Division of Systems Medicine, Imperial College, London, SW7 2AZ United Kingdom

**Keywords:** Biomedical materials, Morphogen signalling

## Abstract

Bone morphogenetic proteins (BMPs) are multifunctional cytokines of the transforming growth factor β (TGFβ) superfamily with potential therapeutic applications due to their broad biological functionality. Designing BMP mimetics with specific activity will contribute to the translational potential of BMP-based therapies. Here, we report a BMP9 peptide mimetic, P3, designed from the type I receptor binding site, which showed millimolar binding affinities for the type I receptor activin receptor like kinase 1 (ALK1), ALK2 and ALK3. Although showing no baseline activity, P3 significantly enhanced BMP9-induced Smad1/5 phosphorylation as well as *ID1*, *BMPR*2, *HEY1* and *HEY*2 gene expression in pulmonary artery endothelial cells (hPAECs), and this activity is dependent on its alpha helix propensity. However, in human dermal microvascular endothelial cells, P3 did not affect BMP9-induced Smad1/5 phosphorylation, but potently inhibited ALK3-dependent BMP4-induced Smad1/5 phosphorylation and gene expression. In C2C12 mouse myoblast cells, P3 had no effect on BMP9-induced osteogenic signalling, which is primarily mediated by ALK2. Interestingly, a previously published peptide from the knuckle region of BMP9 was found to inhibit BMP4-induced Smad1/5 phosphorylation. Together, our data identify a BMP9-derived peptide that can selectively enhance ALK1-mediated BMP9 signalling in hPAECs and modulate BMP9 and BMP4 signalling in a cell type-specific manner.

## Introduction

Bone morphogenetic proteins (BMPs) are a group of multi-functional growth factors within the transforming growth factor β (TGF-β) superfamily, originally discovered due to their potent bone-inducing activity^1^. BMPs have also been shown to play critical roles in embryogenesis and development as well as the maintenance of adult tissue homeostasis, including bone, cartilage, muscle, kidney and blood vessels^2^. Such biological activities have led to great interest in the development of clinical therapies targeting BMP signalling complexes. Recombinant human BMP2 and BMP7 have received approval from the United States Food and Drug Administration (FDA) for treating open tibial shaft fractures and long bone non-unions respectively. Since then, many clinical reports have been published demonstrating the efficacy of BMPs in restoring many types of bone defects in man^3,4^. However, in order to achieve the effective osteoinductive activity, a high dosage of BMPs must be administrated to patients, resulting in high costs. In addition, it has been reported that the production of autoantibodies due to the administration of allogenic or xenogenic BMPs may hinder the use of BMPs in clinical applications^5,6^. Furthermore, large biological molecules like recombinant BMPs exhibit early burst release and degradation problems when incorporated with carriers in a biological environment. These drawbacks have stimulated interest in the development of more cost-effective BMP-based therapies.

**Figure 1:**
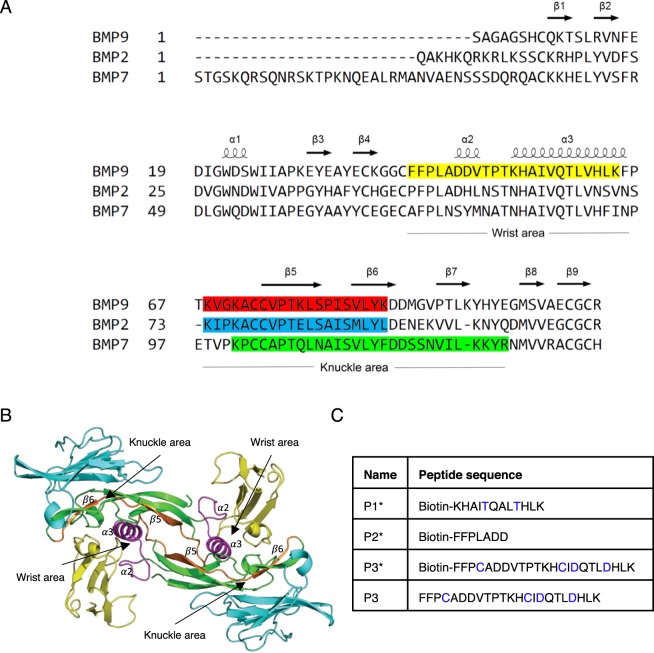
Design of BMP peptides. (**A**) Sequence alignment of BMP9, BMP2 and BMP7 with the secondary structures, “knuckle area” and “wrist area” annotated. Previously reported BMP peptides are highlighted in red, blue and green. P3 sequence in the current study is highlighted in yellow. (**B**) The crystal structure of ALK1:BMP9:ActRIIb (4FAO)^8^. BMP9 in green, ALK1 in yellow, ActRIIb in cyan. The P3 peptide, which is designed from the wrist area of the ALK1-binding surface, is highlighted in magenta. The P4 peptide (Fig. 3), which stretches across the knuckle surface of the BMP9 is highlighted in orange. (**C**) The peptide sequences of P1*, P2*, P3* and P3.

BMPs are homo-dimers, functioning through serine/threonine kinase receptors, specifically forming signalling complexes with two copies of type I receptors (activin receptor-like kinase 1 (ALK1), ALK2 and ALK3) via its “wrist” area and two copies of type II receptors (BMP receptor type II (BMPRII), activin type II receptor a and b (ActRIIa and ActRIIb)) via its “knuckle” area^7,8^ (Fig. 1A,B). Over the past decade, a number of studies have demonstrated peptides designed from the BMP2 knuckle epitope (residues 73–92, Fig. 1A) possess ectopic bone formation activity *in vivo* and can enhance osteogenic signalling in the multipotent mesenchymal cell line (C3H10T1/2)^9,10^. Short peptides from the knuckle area of BMP7 (Fig. 1A) have been shown to promote proliferation and calcium deposition in osteoblasts^11,12^, and a cyclised peptide, THR-123, optimised from the same knuckle area of BMP7, was shown to reverse established fibrosis in murine models of acute and chronic kidney injury in an ALK3-dependant manner^13^.

BMP9 is a potent osteogenic factor, but unlike other osteogenic BMPs, its activity is not inhibited by BMP3 or noggin^14,15^. Long *et al*. showed that BMP9 can reverse pulmonary arterial hypertension (PAH) via selective restoration of BMPRII pathways in several preclinical rodent models^16^. Peptides designed based on the BMP9 knuckle area (Fig. 1A,B) have been shown to induce differentiation of murine preosteoblasts (MC3T3-E1 cells) and cholinergic differentiation in human SH-SY5Y neuroblastoma cells^17,18^. In this study, we describe the design and characterisation of a novel BMP9-mimetic peptide from the ALK1-binding surface (the “wrist” area) that can specifically enhance BMP9-induced Smad1/5 phosphorylation in human pulmonary artery endothelial cells (hPAECs), while inhibit BMP4-induced Smad1/5 phosphorylation in human dermal microvascular endothelial cells (HMEC-1). Our results emphasise the context-dependent nature of BMP signalling, which needs to be taken into account when developing BMP mimetics for therapeutic applications.

## Results

### Design of BMP9 peptides

Peptides P1*, P2*, P3* and P3 (* indicates biotinylation at the N-terminus) were designed from the ALK1-binding site of BMP9 or BMP10 based on the crystal structure of the ALK1:BMP9:ActRIIb complex^8^ (Fig. 1B). Peptide P1* is BMP10-based, whereas P2* and P3* are BMP9-based. Two cysteine residues were introduced in P3 and P3* to allow the possible stabilisation of the peptide by forming a disulphide bond. In addition, two buried valine residues in the BMP9 or BMP10 were changed to threonine (P1*) or aspartic acid (P3* and P3) in the peptides in order to increase the peptide solubility and avoid aggregation (Fig. 1C, highlighted in blue).

### The BMP9 peptide from the ALK1-binding surface enhances BMP9-induced Smad1/5 phosphorylation in hPAECs

**Figure 2:**
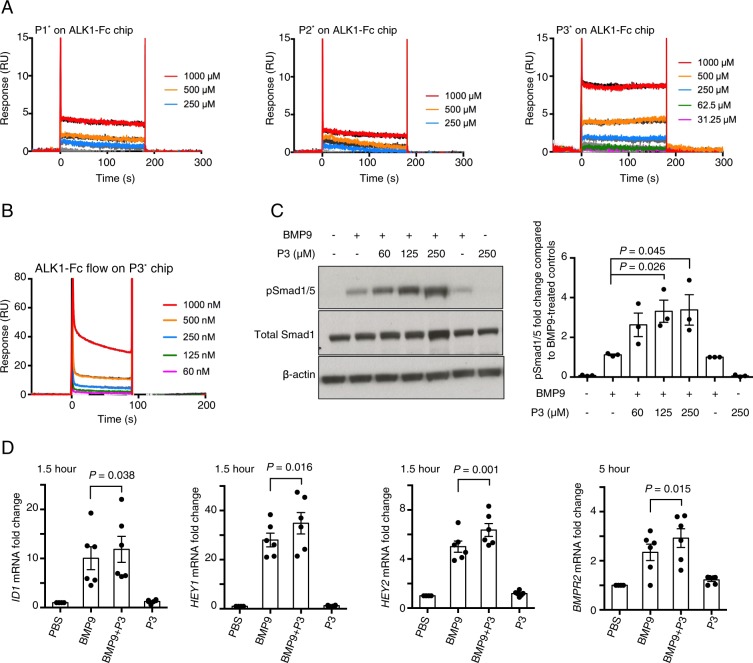
BMP9 peptide P3 is a potentiator for BMP9 signalling in PAECs. (**A**) SPR sensorgrams of peptide P1*, P2* and P3* binding to ALK1-Fc on CM5 chip. Raw data were fitted to steady state binding kinetics to obtain the *K*_*D*_ values of 25M, 84.7 M and 2 mM for P1*, P2* and P3*, respectively. (**B**) SPR sensorgrams of ALK1-Fc binding to peptide P3* on SA chip. The steady state fit yielded a *K*_*D*_ value of 30 mM. (**C**) Representative immunoblots against pSmad1/5, and total Smad1, of the protein extracts from PAECs that have been treated with BMP9 (at 0.03 ng/ml, or 1.24 pM growth factor domain (GFD) dimer) in the presence or absence of P3. Right: quantification of the pSmad1/5 bands by densitometry, N = 3. Paired t-test. (**D**) Effect of peptide P3 (at 160 μM) on BMP9 (at 0.3 ng/ml, or 12.4 pM GFD dimer)-induced gene expression of *ID1*, *HEY1* and *HEY2* following 1.5-hour treatment, or expression of *BMPR2* following 5-hour treatment, in hPAECs. N = 6, data shown as means ± S.E., paired t-test.

We first investigated the binding affinity of these three biotinylated peptides for ALK1-Fc using Surface Plasmon Resonance (SPR). Each peptide was individually passed over a Biacore CM5 chip pre-immobilised with ALK1-Fc. Since all peptides displayed very fast rates for both association and dissociation, the dissociation constants (*K*_*D*_s) were determined using a steady-state fit. As shown in Fig. 2A, P3* has a *K*_*D*_ value of 2 mM against ALK1-Fc whereas P1* and P2* have *K*_*D*_s of 25 and 84.7 M, respectively. The binding between P3* and ALK1-Fc was confirmed by flowing ALK1-Fc over a Streptavidin Biacore chip pre-coated with P3* (Fig. 2B), which gave a *K*_*D*_ value of 30 mM.

With the generation of a milli-molar affinity peptide, we went on to assess the effect of P3 on BMP9 signalling. We initially carried out a Smad1/5 phosphorylation assay in hPAECs with both the biotinylated and the non-biotinylated versions of P3, which gave rise to the same results. The non-biotinylated P3 was used in all subsequent experiments. Interestingly, when P3 was combined with BMP9, the BMP9-induced pSmad1/5 signalling was significantly enhanced by P3 peptide in a dose-dependent manner (Fig. 2C). P3 alone did not induce Smad1/5 phosphorylation in PAECs. Consistent with this, at the mRNA level, several BMP9-regulated target genes, including *ID1*, *BMPR2*, *HEY1 and HEY2*, were significantly enhanced when PAECs were treated with P3 and BMP9 together compared with treating with BMP9 alone (Fig. 2D and Supplemental Fig. 1).

### Secondary structure but not the side chain in the BMP9 peptide is important for its activity

**Figure 3:**
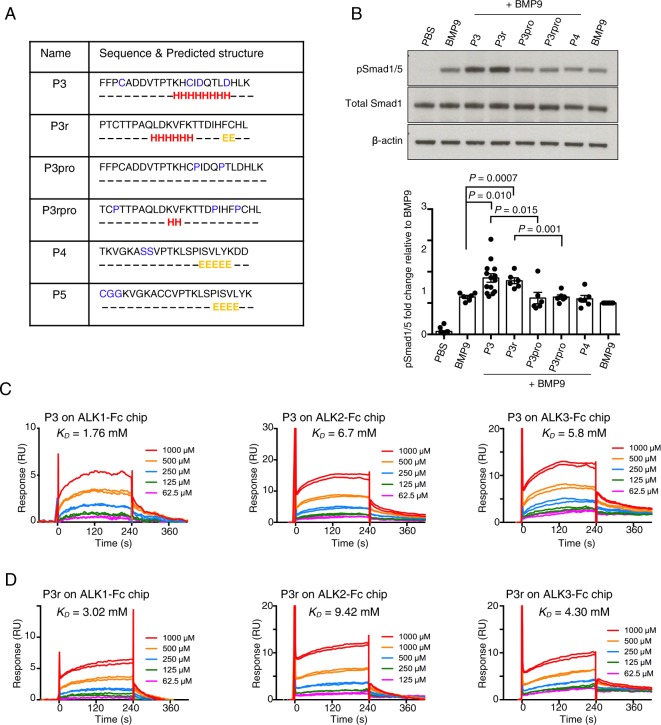
Secondary structure, not the side chains, in BMP9 peptide is important for its activity in hPAECs. (**A**) Sequences of control peptides, with predicted secondary structure using Jpred 4. Amino acids predicted to belong to α-helix or β-sheet are labelled H and E, respectively. In P3, P4 and P5, amino acids that are not identical to the original BMP9 sequences are highlighted in blue. (**B**) Representative immunoblots against pSmad1/5 and total Smad1 of protein extracts from hPAECs treated with peptide P3, P3r, P3pro, P3rpro or P4 (at 250 μM) in the presence of BMP9 (at 0.03 ng/ml, or 1.24 pM GDF dimer). Quantification of the pSmad1/5 bands are shown below. Data shown as means ± S.E., unpaired t-tests were performed on all the peptide + BMP9 treatments against the BMP9 alone treated control, BMP9 + P3 versus BMP9 + P3pro, and BMP9P3r versus BMP9 + P3rpro. Only those with *P* values < 0.05 are shown. A separate BMP9-treatment was included (last lane) for normalisation and not used for statistical analysis. (**C**,**D**) SPR sensorgrams of peptide P3 (**C**) and P3r (**D**) binding to ALK1-Fc, ALK2-Fc and ALK3-Fc on CM5 chip. *K*_*D*_ values obtained from steady state fit are shown on the graphs.

In order to understand how the interaction between peptide P3 and ALK1 allows the stimulation of BMP9-induced pSmad1/5 signalling, we generated several control peptides to test alongside P3 (Fig. 3A). P3 was designed from the BMP9 sequence, which consists of the α3-helix and the pre-helix loop region (which sometimes also forms a short helix such as α2 in the pro-BMP9 structure^19^) (Fig. 1A). Secondary structure analysis using the online server JPred4^20^ also showed that P3 may form an α-helix structure in the middle of its sequence. Therefore, we designed the following control peptides: (1) Peptide P3r was designed by randomly shuffling the amino acid sequence of peptide P3 to destroy the side chain specificity but keep the same set of amino acids, allowing helical secondary structure to form; (2) Peptides P3pro and P3rpro were designed by relocating or inserting prolines in P3 and P3r, which disrupts the α-helical secondary structure, but otherwise shares most of the sequence with P3 and P3r (Fig. 3A); (3) P4 which is a peptide from the knuckle area having the same length as P3 with two Cys mutated to Ser. It is worth noting that knuckle peptides based on BMP2^9^, BMP7^13^ or BMP9^17,18^ have been shown to possess BMP osteogenic activity on their own.

Signalling assays in hPAECs showed that, similar to P3, P3r significantly enhanced BMP9-induced phosphorylation of Smad1/5. The knuckle peptide P4 had negligible effects on BMP9 signalling in this assay (Fig. 3B). Introducing two prolines into P3 and P3r in the α-helix region significantly decreased the effect on BMP9-induced Smad1/5 phosphorylation. P3 and P3r are likely to adopt similar alpha-helical structures whereas this is not possible for P3pro, P3rpro or P4. This suggests that the enhancement of BMP9-induced pSmad1/5 phosphorylation in hPAECs is not due to the specific amino acid side chain interactions, but is likely due to backbone-mediated interactions or global shape complementation conferred by the secondary structure. Indeed, an SPR study detected similar millimolar range weak binding of peptide P3 and P3r to both ALK1, ALK2 and ALK3 (Fig. 3C,D), while P3pro, P3rpro and P4 showed negligible binding (Supplemental Fig. 2), indicating that the interactions between the peptides and the type I receptors were most likely dependent on the secondary structure rather than amino acid side chain specificity.

### The effect of peptide P3 is cell type dependent

**Figure 4:**
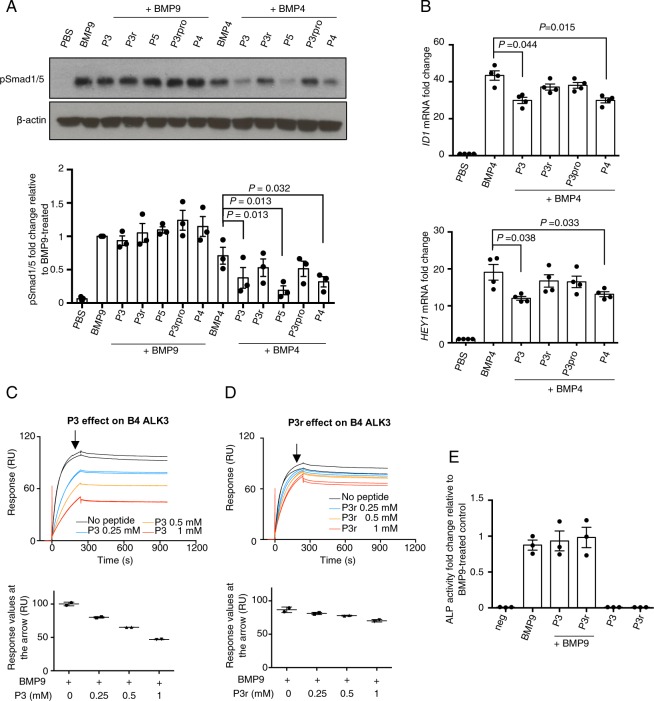
The effect of peptide P3 is BMP and cell type-dependent. (**A**) Representative pSmad1/5 immunoblot of protein extracts from HMEC-1 treated with peptide P3, P3r, P3rpro P4 and P5 (at 250 μM) with or without BMP9 (at 0.03 ng/ml, or 1.24 pM GFD dimer) or BMP4 (at 0.3 ng/ml, or 11.54 pM GFD dimer). Quantification of pSmad1/5 from three independent experiment is shown below. N = 3. Data shown as means ± S.E. In the BMP4-treated group, paired t-tests against BMP4-treated control were performed, and those with *P* values < 0.05 are shown. (**B**) Effect of peptide P3 and control peptides (at 160 μM) on BMP4 (3 ng/ml, or 115.4 pM GFD dimer) stimulated gene induction of *ID1* and *HEY1* in HMEC-1, N = 4. Data shown as means ± S.E., paired t-tests against BMP4-treated control, and those with *P* values < 0.05 are shown. (**C**,**D**) SPR sensorgrams of BMP4 binding to ALK3-Fc in the presence of peptide P3 (**C**) or P3r (**D**) at various concentrations on an ALK3-Fc coated CM5 chip. Plots of the maximum binding levels (indicated by the arrows) against peptide concentrations are shown below each graph respectively. (**E**) Effect of peptide P3 and control peptides (at 160 μM) on BMP9-induced ALP activity in C2C12 cells. Data were obtained from three independent experiments, with duplicated wells for each treatment condition in a single experiment. Data shown as means ± S.E.

Since peptide P3 has no specificity against BMP type I receptors, we tested the effect of P3 on BMP signalling which is not ALK1-dependent. In addition, we asked whether the peptide P3 dependent enhancement of BMP9-induced Smad1/5 signalling can be observed in other cell types. The human endothelial cell line HMEC-1 can mediate both ALK1-depedent BMP9 and ALK3-dependent BMP4 signalling^21^, therefore we performed signalling assays in these cells. In the presence of peptide P3 and the control peptides, there was no significant change observed for BMP9-induced Smad1/5 phosphorylation. In contrast, the BMP4-induced signalling was significantly suppressed by the addition of peptide P3, which could be observed by both Smad1/5 phosphorylation, and the induction of *ID1* and *HEY1* mRNA (Fig. 4A,B). Interestingly, P3r, unlike P3, failed to inhibit BMP4 signalling in HMEC-1. The SPR data demonstrated the maximum binding of BMP4 to ALK3-Fc is significantly reduced in the presence of P3 in a dose-dependent manner, with maximum binding decreased by nearly 50% when P3 was added at 1 mM concentration (Fig. 4C). In contrast, compared with P3, P3r was much less effective in competing BMP4 binding to ALK3 (Fig. 4D). The knuckle peptide P4 also showed suppression of BMP4-induced Smad1/5 phosphorylation and BMP4-stimulated downstream gene expression of *ID1* and *HEY1* in HMEC-1 cells (Fig. 4A,B). Another knuckle peptide P5, which is a variant of P4 (Fig. 3A) and has the identical sequence to the published studies^22^, also significantly suppressed BMP4-induced Smad1/5 phosphorylation in HMEC-1 cells (Fig. 4A). Interestingly, P3 peptide does not have any effect on BMP6 signalling in HMEC-1 cells (Supplemental Fig. 3). Consistent with observations in hPAECs, the peptides alone did not display any signalling activity in HMEC-1, either by Smad1/5 phosphorylation or mRNA gene expression (data not shown).

BMP9 signalling in PAECs is mediated by ALK1, however it can also induce alkaline phosphatase (ALP) activity in mouse myoblast cells (C2C12) via a low affinity receptor ALK2. We next tested how peptide P3 and the control peptides affect the non-ALK1 mediated BMP9 signalling, such as in C2C12 cells. No difference in ALP activity was observed when peptides P3 or P3r were added together with BMP9 compared with BMP9 treatment alone (Fig. 4E).

## Discussion

In recent years, studies have advanced our knowledge of cellular and systemic functions of BMPs; and BMP-based therapeutic treatments are being developed for cardiovascular and kidney diseases^13,16^. The identification of BMP agonists or BMP mimetics remains an attractive strategy due to the high cost and difficulty in manufacturing large quantities of clinical grade BMPs. Peptides that are designed from the knuckle area of BMP2, BMP7 and BMP9 were found to display similar osteogenic activity to the corresponding BMP molecule^9–12^. Furthermore, peptides from the prodomain of BMP7, namely BFP-1/2/3, were also shown to induce stronger alkaline phosphatase activity in multipotent bone marrow stromal cells (MBSCs)^23–25^. In addition, a BMP2 knuckle peptide has been shown to possess similar bone inducing activity *in vivo* to BMP2, but did not induce side effects as observed for BMP2^26^, therefore BMP peptides hold the potential for improving specificity. Although these findings are exciting, very few studies have performed in-depth binding and mechanistic investigation on how these peptides achieve their specificity. Intriguingly, a cyclised BMP7 knuckle peptide THR123 was reported to successfully reverse established kidney fibrosis in mouse models of acute and chronic renal injury^13^, however, the mechanism of action of THR123 has been challenged^27,28^. THR123 has been advanced to a randomised, double-blinded, placebo-controlled, multidose clinical trial in 452 patients with high risk of acute kidney injury after cardiac surgery, and has shown lack of efficacy in all the measured endpoints^29^. Understanding the mechanisms of action will aid the future design of efficacious BMP-based peptides.

In the current study, we identified a BMP9 mimetic peptide P3, designed from the “wrist area” of BMP9, which enhanced BMP9-induced Smad1/5 phosphorylation selectively in hPAECs but inhibited BMP4-induced Smad1/5 phosphorylation in HMEC-1. To gain more insight into the mechanisms behind the activity of peptide P3, we designed control peptides P3r, P3pro and P3rpro which allowed the investigation of how sequence specificity and secondary structure of P3 contribute to its function. We found that disruption of the secondary structure of P3 and P3r via proline insertions leads to a complete loss of their *in vitro* activity against BMP9/BMP4 and binding affinity towards the type I receptors. This suggests that the enhancement of BMP9 activity by P3 is likely due to a weak backbone-mediated interaction between peptide P3 and the type I receptors on the surface of hPAECs, which is supported by the millimolar binding affinity towards ALK1 (Fig. 3C) and a trend of P3 to increase the maximum binding of BMP9 to ALK1 (Supplemental Fig. 4). The different expression levels of ALK1 on the cell surface of hPAEC compared to HMEC-1 and C2C12 might explain why peptide P3 has no effect on BMP9 activity in HMEC-1 and C2C12. The inhibition of BMP4 activity in HMEC-1 is likely due to the more specific interactions between peptide P3 and ALK3, since loss of sequence specificity diminished the peptide activity in P3r and P3pro. This is also supported by the SPR study where P3 is much more effective than P3r in blocking BMP4 binding to ALK3-Fc (Fig. 4C,D).

It is intriguing why the same peptide could promote BMP9 signalling in PAECs while inhibit BMP4 signalling in HMECs, an effect which is also recapitulated in the SPR binding studies (Fig. 4A–D, Supplemental Fig. 4). Whilst it is difficult to provide a direct structural explanation, this may relate to the difference in affinities and concentrations of the receptors for the ligands. ALK1 binds BMP9 with affinity of around 0.03 nM^8^. The reported *K*_*D*_ values of BMP4 for ALK3 (with ALK3 expressed from different host cells) are 1–10 nM^30,31^. It is plausible that the inhibitory effect of P3 on BMP4 signalling in HMECs is due to P3 competing the BMP4 binding site on ALK3 (Fig. 4C). The situation with PAECs may be more complicated. Not only do PAECs express high levels of ALK1, they also express high levels of endoglin at cell surface which can capture and orientate BMP9 for optimum signalling^32,33^. The slight enhancement of maximum BMP9 binding to ALK1-Fc is in agreement with the signalling result in PAECs, but is difficult to explain from a structural or protein-protein interaction perspective. Of note, control experiment shows that P3 does not bind to BMP9 (Supplemental Fig. 5). One possibility is that P3 (and P3r) binding to ALK1 allows ALK1 to adopt a conformation that favours the binding to BMP9. Further studies are required to understand how P3 enhances BMP9 signalling in PAECs and evaluate its effect on other primary endothelial cells.

In our study, the effects of P3 were only observed in the presence of BMP9 or BMP4, not on its own in the serum-free medium. It is worth noting that many previous studies reporting the osteogenic potential of knuckle region peptides alone were carried out either *in vitro* in the presence of serum, or in *in vivo* animal models, where BMP9 will be present at active concentrations. It is possible that the activity of some previous knuckle region peptides was also via activating the serum BMP activity rather than mimicking BMP activity. In addition, our observation that knuckle peptides can inhibit BMP4 signalling should be considered when analysing the effect of these knuckle peptides *in vivo*.

Taken together, we have identified a BMP9-based peptide, P3, that acts as a BMP9 potentiator in human PAECs. Interestingly, we also observed the same peptide exerting different effects in a BMP-dependent and cell type-dependent manner. Our data provided insights into the mechanisms underlying the activity of P3, which can guide future development of a BMP9-based agonist for therapeutic purposes.

## Materials and Methods

### Materials

ALK1-Fc, ALK2-Fc and ALK3-Fc were purchased from R&D Systems. Primary antibodies for western blot analysis were purchased from Cell Signalling Technology or CalBioreagents. All peptides used in this study were purchased and synthesised by PepMic (Suzhou, China). HPAECs and endothelial growth medium were purchased from Lonza (Wokingham, Berkshire, UK). HMEC-1 cells were from the Centre for Disease Control (CDC, Atlanta, GA). All other cell culture media were purchased from Life Technologies.

### Surface plasmon resonance (SPR)

Peptide-receptor binding assays were performed using Biacore T200 biosensor (Biacore/GE Healthcare). ALK1-Fc, ALK2-Fc or ALK3-Fc was covalently linked onto individual flow cells of a research grade CM5 chip (GE Healthcare) via amine-coupling using standard manufacturer’s protocol. One flow cell was used as a control for subtracting non-specific binding. Peptides were injected over the immobilised chip at various concentrations up to 1 mM at a flow rate of 30 μl/min for 3 min at 37 °C in running buffer containing 0.01 M HEPES, 0.5 M NaCl, 3 mM EDTA and 0.005% v/v Surfactant P20 (pH 7.4). At the end of each peptide injection, the chip surface was regenerated with 4 M Guanidine hydrochloride (GnHCl). The biotinylated peptide P3* was immobilised onto a streptavidin (SA) chip (GE Healthcare) following the manufacturer’s protocol. Binding assays were carried out using a similar protocol as described above, only with a different injection time of 4 min. For BMP9-ALK1 and BMP4-ALK3 binding assays, BMP9 and BMP4 were added at 50 nM alone or in the presence of peptide P3 or P3r at 0.25 mM, 0.5 mM or 1 mM. Each cycle was set with a dissociation time of 12 min. Duplicated injections were performed for each ligand along with buffer blanks. The equilibrium dissociation constant *K*_*D*_ was determined by fitting the data to a steady-state model using BIAevaluation software (GE Healthcare). Processed data were plotted in GraphPad Prism.

### Signalling assays monitoring Smad1/5 phosphorylation

Endothelial cells were seeded in 6-cm dishes at 9 × 10^5^ cells/dish and cultured for 24 hours (h) to full confluence, and then serum-starved for 16 h before treatments with control buffer, BMP9 alone, BMP9 with peptides, or peptides alone. After 40 min, cells were washed once with PBS and snap-frozen on dry ice to harvest. Cells were then lysed with buffer containing 125 mM Tris-HCl (pH 6.8), 2% SDS and 10% glycerol. The total protein concentration was determined using the *DC*^TM^ protein assay (Bio-Rad), of which 30 μg of total cell proteins was fractionated on 12% SDS-PAGE under non-reducing condition. The phosphorylation of Smad1/5 and total Smad1 were detected by anti-phospho-Smad1/5 antibody (9516 S) and anti-Smad 1 antibody (9743 S). The intensity of pSmad1/5 bands were quantified using ImageJ software, using β-actin or α-tubulin as the loading control.

### Signalling assays monitoring gene expression using quantitative PCR (qPCR)

Endothelial cells were seeded in 6-well plates at 3 × 10^5^ cells/well and serum-starved for 16 h prior to treatment. After 1, 1.5 or 5 hours, cells were washed with PBS and snap-frozen on dry ice. RNAs were extracted using the RNeasy Plus Mini Kit (Qiagen). cDNAs were generated from 1 μg of total RNA by using the High-Capacity cDNA Reverse Transcription Kit (Applied Biosystems) according to the manufacturer instructions. Q-PCR reactions were prepared with 2.8 μl of 10 times diluted cDNA samples and 5.8 μl of master mix containing 5 μl of the SYBR Green Jumpstart Taq ReadyMix (Sigma), 200 nM of each forward and reverse primers and 10 nM of Rox Reference Dye. Specific primers were used for *β*_*2*_*M* (sense 5′-CTCGCGCTACTCTCTCTTTCT-3′; antisense 5′-CATTCTCTGCTGGATGACGTG-3′), *ID1* (sense 5′-GACGGCCGAGGCGGCATG-3′; antisense 5′-GGGGAGACCCACAGAGCACG-3′), *BMPR2* (sense 5′-CAAATCTGTGAGCCCAACAGTCAA-3′; antisense 5′-GAGGAAGAATAATCTGGATAAGG-ACCAAT-3′), *HEY1* (sense 5′-AGCTAGAAAAAGCCGAGATCCTGCA-3′; antisense 5′-CCCGAAATCCCAAACTCCGATAGTC-3′), *HEY2* (sense 5′-ACCATCGACGTGGGGAGCGA; antisense 5′-ATCCGATCCCGACGCCTTTTC-3′). Reactions were amplified on a QuantStudio 6 Flex RT-PCR instrument (Applied Biosystems). The relative expression of target mRNAs was normalised to house-keeping gene *β*_*2*_*M* using the ΔΔCT method, displayed as the fold-change relative to control samples, and shown as means ± S.E. where applicable.

### ALP assay

C2C12 mouse myoblasts were seeded in 24-well plates at 25,000 cells/well with antibiotics for 48 h. Treatments were performed in DMEM, 0.25% FBS with antibiotics in a final volume of 1.1 ml for 65 h. Cells were harvested and lysed in 1% Triton-X100 in PBS and the extracted total protein was quantified by Bio-Rad *DC*^TM^ protein assay (Bio-Rad). ALP activity was determined by incubating 15 μg of the total protein with ALP substrate *p*-nitrophenyl phosphate (PNPP, Sigma-Aldrich) and absorbance of the product *p*-nitrophenol (PNP) was recorded at 405 nm in 5 min intervals for 1 h. The relative ALP activity was expressed as fold change to the BMP9-treated control with data presented as means ± S.E.

### Statistical analysis

Analyses were performed using GraphPad Prism. All data were presented as means ± S.E. and student t tests were used for comparison between two groups as detailed in the figure legends. *P* < 0.05 was considered statistically significant, and the values are shown on the graphs.

## Supplementary information

Supplemental Figures and Figure legends
