# Supplementary material for: A Bone Morphogenetic Protein (BMP)-derived Peptide Based on the Type I Receptor-binding Site Modifies Cell-type Dependent BMP Signalling
Source: Sci Rep. 2019 Sep 17;9:13446. doi: 10.1038/s41598-019-49758-x (PMC6748948; doi:10.1038/s41598-019-49758-x)
Supplement: Supplementary file 1 — Supplemental Figures and Figure legends [file 41598_2019_49758_MOESM1_ESM.pdf]

A Bone Morphogenetic Protein (BMP)-derived Peptide Based on the Type I Receptor-binding Site Modifies Cell-type Dependent BMP Signalling

Zhen Tong[1], Jingxu Guo[1], Robert C. Glen[2,3], Nicholas W Morrell[1] and Wei Li[1,*]

1. The Department of Medicine, University of Cambridge School of Clinical Medicine, Cambridge, CB2 0QQ, United Kingdom; 2. Department of Chemistry, University of Cambridge, CB2 1EW; 3. Computational and Systems Medicine, Imperial College, London, SW7 2AZ, United Kingdom.

Supplemental figures and figure legends.

Supplemental Figure 1. Effect of peptide P3 on BMP9 induced gene expression at 1 hr in hPAECs.

Supplemental Figure 2. SPR sensorgrams of peptide P3pro, P3rpro and P4 binding to ALK1-Fc, ALK2-Fc and ALK3-Fc on CM5 chip.

Supplemental Figure 3. P3 does not modify BMP6 signalling in HMEC-1 cells.

Supplemental Figure 4. Effects of P3 and P3r on BMP9 binding to ALK1-Fc.

Supplemental Figure 5. SPR binding of P3 to a BMP9 coated CM5 chip.

Supplemental Figure 1

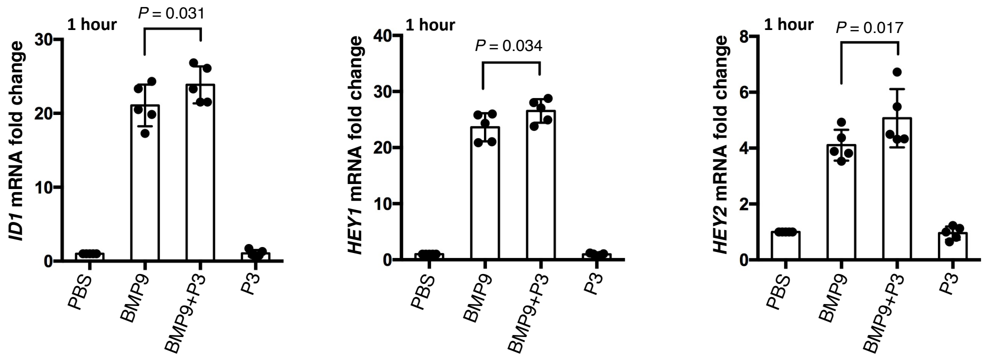

Supplemental Figure 1. Effect of peptide P3 on BMP9-induced gene expression at 1 hr in hPAECs. PAECs were treat with BMP9 (at 0.3 ng/ml, or 12.4 pM GFD dimer) in the presence or absence of peptide P3 (160 μM) for 1 hour before cells were harvested for RNA extraction and qPCR analysis. N=5. Data shown as means ± S.E., two tailed, paired t-test.

Supplemental Figure 2

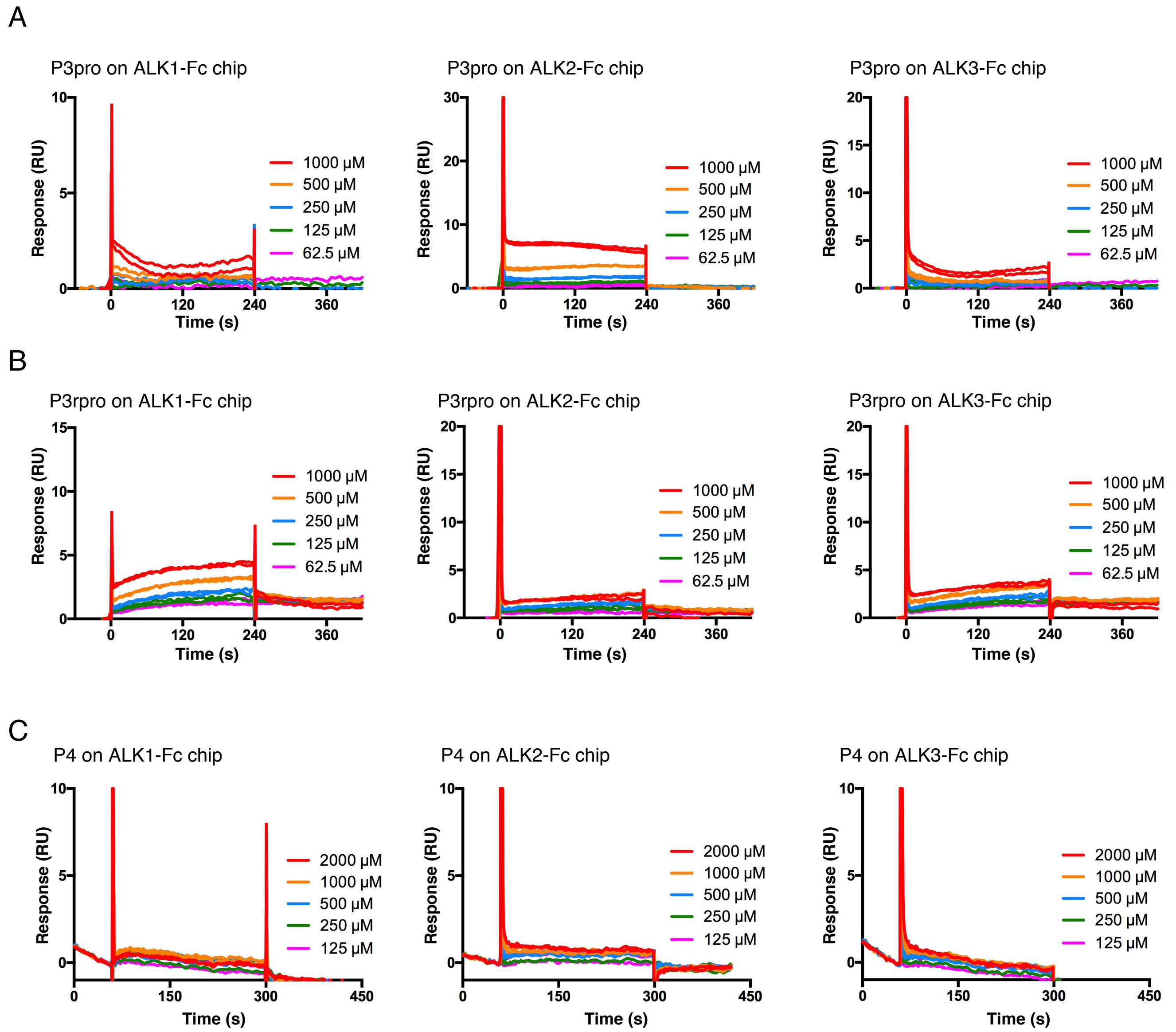

Supplemental Figure 2. SPR sensorgrams of peptide P3pro, P3rpro and P4 binding to ALK1-Fc, ALK2-Fc and ALK3-Fc on CM5 chip.

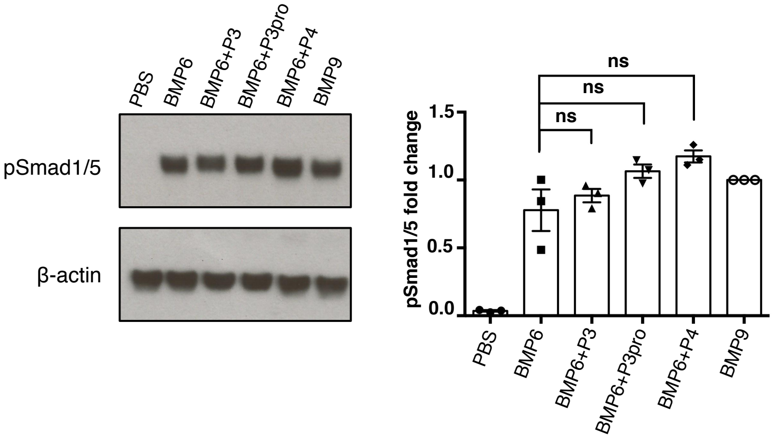

Supplemental Figure 3. P3 does not modify BMP6 signalling in HMEC-1 cells. Representative immunoblots against phospho-Smad 1/5. Protein extracts from HMEC-1 that have been treated with PBS, BMP6 (10 ng/ml) with or without peptides (250 μM) for 40 min. BMP9 (0.03 ng/ml) was included as a reference. Data are shown as means ± S.E., two-tailed, paired t-test.

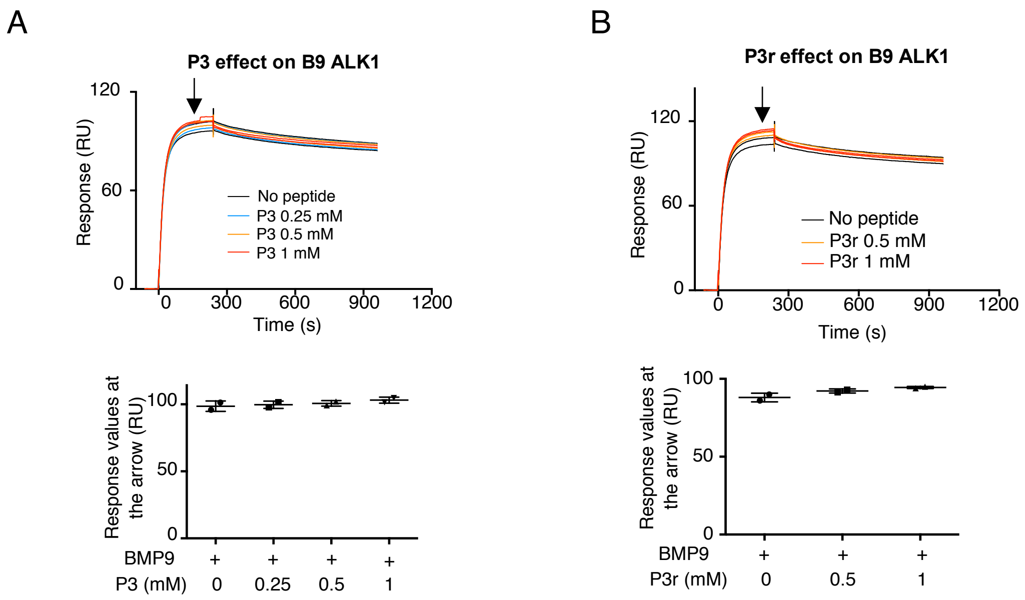

Supplemental Figure 4. Effects of P3 and P3r on BMP9 binding to ALK1-Fc. (A) SPR sensograms of BMP9 binding to ALK1-Fc in the presence or absence of peptide P3. Below: Plot of the maximum binding level of BMP9 on ALK1-Fc chip (indicated by the arrow) with P3 peptide at 0, 0.25, 0.5 or 1 mM. (B) SPR sensograms of BMP9 binding to ALK1-Fc in the presence or absence of peptide P3r. Below: Plot of the maximum binding level of BMP9 on ALK1-Fc chip (indicated by the arrow) with P3r peptide at 0, 0.5 or 1 mM.

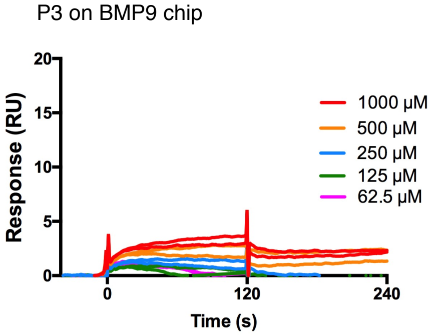

Supplemental Figure 5. SPR binding of P3 to a BMP9 coated CM5 chip. BMP9 (R&D system) was immobilised onto a research grade CM5 chip (GE Healthcare) via amine-coupling using standard manufacturer's protocol. BMP9 immobilised surface activity was verified by flowing Alk1-Fc. One flow cell was used as reference. Peptide P3 was injected over BMP9 surface at various concentrations up to 1 mM at a flow rate of 30 µl/min for 2 min at 37 °C in running buffer containing 0.01 M HEPES, 0.5 M NaCl, 3 mM EDTA and 0.005 % v/v Surfactant P20 (pH 7.4). At the end of each cycle, surface was regenerated using 2 M Guanidine hydrochloride (GnHCl). Duplicated injections were performed for each ligand along with buffer blanks. Steady state fit by the Biacore evaluation programme gives a K_D value of 24 M.
